# Towards universal coverage for nutrition services in children under five years—A descriptive analysis of the capacity of level one hospitals to provide nutrition services in five provinces of Zambia

**DOI:** 10.1371/journal.pone.0232663

**Published:** 2020-05-12

**Authors:** Kutha Banda, Sandra Chilengi-Sakala, Chipwaila Choolwe Chunga, Hiwote Solomon, Victor Chalwe, Justine Mweene Nkaama, Colleen Leonard, Mupeta Bobo, Agness Aongola, Godfrey Biemba

**Affiliations:** 1 National Health Research Authority, Ministry of Health, Lusaka, Zambia; 2 Doctor of Public Health Program, Boston University School of Public Health, Boston, Massachusetts, United States of America; 3 Department of Public Health, Ministry of Health, Lusaka, Zambia; Tulane University School of Public Health and Tropical Medicine, UNITED STATES

## Abstract

**Background:**

Malnutrition continues to be a major public health challenge in Zambia. To effectively address this, health systems must be well strengthened to deliver an effective continuum of care. This paper examines health systems issues and services in relation to nutritional support to children under five years, in order to identify gaps and propose interventions towards universal coverage of essential nutrition services.

**Methods:**

This analysis utilized data from a cross sectional mixed-methods study on factors associated with Severe Acute Malnutrition (SAM) in under-five children to assess health facility nutrition services on offer at select level-one hospitals in five out of ten provinces in Zambia. Stata version 13 was used for analysis. We conducted univariate analysis to assess nutrition services offered, functionality of equipment and tools, availability of human resource and human resource development, and availability of drugs used for assessment and management of nutrition-related health outcomes.

**Results:**

We found large variations in the level of nutrition services on offer across districts and provinces. Eighty-eight percent of all the hospitals sampled provided group nutrition counseling and 92% of the hospitals in our sample offered individual nutrition counseling to their clients. Overall, the existence of referral and counter-referral systems between the Community Based Volunteers and hospitals were the lowest among all services assessed at 48% and 58% respectively. We also found inadequate numbers of human resource across all cadres with an exception of nutritionists as recommended by the Ministry of Health.

**Conclusions:**

This study has revealed a number of gaps in the health system and health service delivery that requires to be addressed; most notably, a lack of tools, policies and guidelines, drugs and health specialists to help care for malnourished infants and children. Our findings also reveal inadequate referral systems between the community and health facilities in the management of severe acute malnutrition. Achieving universal coverage for nutrition services in Zambia will require a lot more attention to the health systems issues found in this study.

## Introduction and rationale

Malnutrition remains a global public health challenge, especially among children under five years, with close to half (45%) of all deaths in this age group linked to malnutrition [1]. In 2018, a joint report by the United Nations Children’s Fund (UNICEF), World Health Organization, and the World Bank highlighted the slow progress that has been made in reducing the prevalence of undernutrition in under-5 children [2]. Globally the prevalence of under-5 children who were stunted, wasted, or overweight were 21.9%, 7.3%, and 5.9%, respectively.

In 2016, the United Nations (UN) General Assembly declared 2016 to 2025 the UN Decade of Action on Nutrition [1]. This Decade of Action called for policy actions across six areas including aligning health systems to nutrition needs and providing universal coverage of essential nutrition interventions.

The joint report by the United Nations Children’s Fund (UNICEF), World Health Organization, and the World Bank report found 58.8 million under-5 children living in Africa were stunted, 14 million were classified as wasting, and 9.5 million were overweight [2]. Eastern and Central Africa bear the greatest burden of stunted children under the age 5 (35% and 32%, respectively) [3]. Western and Northern Africa both had the highest percentage of under-five children classified as wasted (8% and 9%, respectively). When assessing the prevalence of overweight children under-5, Southern Africa had the greatest burden in Africa with 13% of under-5 children having medium levels of overweight.

Although the UNICEF/WHO/World Bank report [3] found that Southern Africa had relatively low levels of stunting and wasting, Zambia has one of the highest rates of malnutrition. The 2013–14 Zambia Demographic and Health Survey (ZDHS) found that 40% of children under five in Zambia were stunted, indicating chronic malnutrition across the country [4]. This was most common in Northern Province with more than 50% of children affected by stunting, compared to 36% in Lusaka, Copperbelt, and Western Provinces. Wasting was less common in Zambia with a prevalence of only 6%. Additionally, the 2013–14 ZDHS found that 15.4% of children less than 6 months old suffered from acute malnutrition [4]. While trends have generally improved when comparing the 2013–14 ZDHS results with the 2001–02 and 2007 ZDHS’, malnutrition remains a major problem in Zambia and health facilities have to be equipped to provide treatment.

In Zambia, the Ministry of Health successfully applied for financial assistance from the World Bank under the Zambia Health Services Improvement Project (ZHSIP) in 2014 in an effort to among other things improve the nutrition status of children under five. This is a five-year project to strengthen health systems and improve health service delivery with a focus to improve Reproductive, Maternal, Neonatal and Child Health (RMNCH) and Nutrition outcomes in the five poorly performing provinces, namely: Luapula, Muchinga, Northern, North-Western, and Western Provinces for five years starting in 2015. This study therefore, was anchored under this project and this paper aims to describe the nutrition services on offer to children under-five presenting with severe acute malnutrition in selected first level hospitals in an effort to identify gaps in the health system in providing universal essential nutrition services. This study was conducted in level 1 hospitals as all hospitals at this level are expected to provide nutrition services following the guidelines discussed in this paper.

## Methods

### Study design and participants

This analysis utilized data from a cross sectional mixed-methods study on factors associated with Severe Acute Malnutrition (SAM) in under-five children to assess health facility nutrition services on offer at select level-one hospitals in five out of ten provinces in Zambia. Data was derived from a facility assessment tool that took stock of the nutrition services available through interviews with staff to understand the nutrition services on offer, as well as the presence of essential guidelines and medicines.

### Sample size consideration

Being a study under the Zambia Health Service Improvement Project (ZHSIP), our sample was derived from the five ZHSIP Provinces namely: Luapula, Muchinga, Northern, North-Western, and Western Provinces. Our sample unit was health facilities in these five Provinces. A complete enumeration of all level one hospitals in each Province was listed and numbered. After the listing, a random number generator using Microsoft Excel was utilized to randomly select each health facility until a sample size of 5 facilities in each province was generated. The sample was restricted to this numberdue to financial and time constraints. Most Provinces have only one level-one hospital per District, thus our sample had one hospital per District (with the exception of Luapula Province which had two level-one hospitals in one of the sampled Districts- Samfya). In total, 25 level-one hospitals were selected for inclusion in the study.

The target population for this study were health care workers in the hospitals. At each sampled health facility, researchers further used purposive sampling to select participants for the facility assessment tool based on their official designation (In-Charge, Pharmacist, Nutritionist, and Nutrition Focal Point Person) who responded to questions specific to their departments/units in the questionnaire.

### Data collection and management

Data collection was conducted by five survey teams from January to February of 2018. All targeted Districts and hospitals were covered with the exception of Chama District in Muchinga Province due to challenges caused by severe flooding and road blockages at time of survey. The health facility assessment utilized the SPRING Tool for Rapid Evaluation of Facility-Level Nutrition Assessment, Counseling, and Support [5]. This tool helps gather information on the capacity of health facilities to implement Nutrition Assessment Counseling Support for pregnant women, children, and people living with HIV (PLHIV). The facility assessment tool had three major components with specific questions for the Head of the hospital, the Pharmacists and the Nutritionist. The tool was first administered to the Head of each hospital to gather general information about the hospital, then to the Pharmacist and Nutritionist or nutrition focal point person to understand services on offer and trainings in provision of nutrition services provided to staff.

### Data analysis

Data were analyzed using STATA 13 (StataCorp, College Station, Texas, USA). We conducted descriptive analysis on demographic information of study participants. Subsequently, demographic information was disaggregated by Province. We then conducted univariate analysis to assess nutritional services offered, functionality of equipment and tools, availability of human resource and human resource development, and availability of drugs used for assessment and management of nutrition-related health outcomes.

### Ethical consideration

Ethical clearance was obtained from the University of Zambia Biomedical Research Ethics Committee. At the facilities, the head of the hospital first provided consent to undertake the study in their hospitals and thereafter, all the other officers interviewed provided individual consent. All participants were required to sign a consent form informing them of the purpose of the study, the benefits and risks of participating, and their right to stop the interview at any point. All participants were also given an additional consent form to keep for their own records.

## Results

### Characteristics of the participants

A total of 73 participants were interviewed for the facility assessment (Table 1). The majority of participants were from Luapula, Northern and North-Western Provinces (21.9%). The role of most respondents were that of the hospital In-Charge and the Pharmacists (34.3%). A total of 25 hospitals were sampled, 21 (84%) of which were Government hospitals.

**Table 1:** Characteristics of respondents.

**Number of the respondents by Province**	**n(%)**
Luapula	16(21.9)
Muchinga	12(16.4)
North-Western	16(21.9)
Northern	16(21.9)
Western	13(17.8)
**Total respondents**	**73(100)**
**Role of the respondents**	**n(%)**
In Charge	25(34.3)
Pharmacist	25(34.3)
Nutritionist	21(28.8)
Nutrition Focal Point Person	2(2.7)
**Total respondents**	**73(100)**
**Facility Management**	**n(%)**
Government	21(84)
Faith-based (NGO)	3(12)
Other (SPECIFY)	1(4)
**Total number of facilities**	**25(100)**

### Nutrition services offered at selected level-one hospitals

Table 2 shows the nutrition services offered at the hospitals, disaggregated by Province. These nutrition services indicators collected were based on the SPRING tool used in the study. Group and individual nutrition counseling were offered at all hospitals in three Provinces except for North-western and Western Provinces, overall, 88% and 92% of all the sampled facilities provided group and individual counseling respectively. Most of the hospitals in Luapula, Northern and Western Province utilized Community-Based Volunteers (CBVs) as part of their care system in the sampled hospitals. Additionally, most hospitals did not have systems put in place for CBVs to offer nutritional services or referrals to other hospitals. Referrals of malnutrition cases to other hospitals were rare in our sample with only four hospitals in Northern Province reporting that they referred malnutrition cases to other hospitals. Overall, the existence of referral and counter-referral systems between the CBVs and hospitals were the lowest among all services assessed at 48 and 58% respectively.

**Table 2:** Nutrition services offered at the hospitals by Province.

Indicator by Province	Luapula (n = 5)	Muchinga (n = 4)	Northern (n = 5)	North-Western (n = 6)	Western (n = 5)	Total # of hospitals providing the service N = 25	Overall % of hospitals providing the service % (n/N)
Hospitals that provide Group nutrition counseling	5	4	5	5	3	22	88%
Hospitals that provide Individual nutrition counseling	5	4	5	5	4	23	92%
Hospitals where Community Based Volunteers (CBV) engaged as part of care system	4	2	4	3	4	17	68%
Hospitals where CBVs provide nutrition services	4	2	4	2	3	15	60%
Hospitals that have a system of community workers/volunteers or other NGO social services refer clients to it	4	2	4	2	2	14	56%
Hospital that have a system for referring clients to any other facility and/or community-based services	2	2	4	2	2	12	48%

### Availability of equipment and tools

The majority of respondents (73.9%) reported that their hospitals had an infant/pediatric scale in the Peadiatric Ward and most of them (52.2%) reported having up-to two functional scales in the hospital, in other words, Table 3 below shows that out of 23 respondents 9 reported that only one scale was functional, while 12 reported that two scales were functional, and only 2 reported having more than 3 scales that were functional. Most hospitals (82.6%) reported having length boards in the hospital to use for nutritional services. The majority of respondents (56.5%) reported having a stadiometer, however, unlike the other equipment, this was not observed/seen at the hospital.

**Table 3:** Availability of equipment and their functionality.

Indicator (N = 23)	Available and observed n (%)	Available not observed n (%)	Not available n (%)	Number of equipment functional		
				1 n (%)	2 n (%)	3+ n (%)
Infant/pediatric scale	17 (73.9)	6 (26.1)	0	9(39.1)	12(52.2)	2(8.7)
Adult weighing	17 (73.9)	6 (26.1)	0	10 (43.5)	6(26.1)	7(30.4)
Length board (length/height)*	19 (82.6)	4 (17.4)	0	11 (50)	9(40.9)	2(9.1)
Stadiometer*	10 (43.5)	13 (56.5)	0	9 (75)	3(25)	0
MUAC tape for children*	21 (91.3)	0	2 (8.7)	1 (4.8)	2(9.5)	16(76.2)
MUAC tape for pregnant and lactating women*	13 (56.5)	10 (43.9)	0	4 (30.8)	2(15.4)	7(53.9)

*N for indicator ‘number of equipment functional not equal to 23 due to some missing responses

### Protocols and guidelines for providing nutrition services

**Fig 1:**
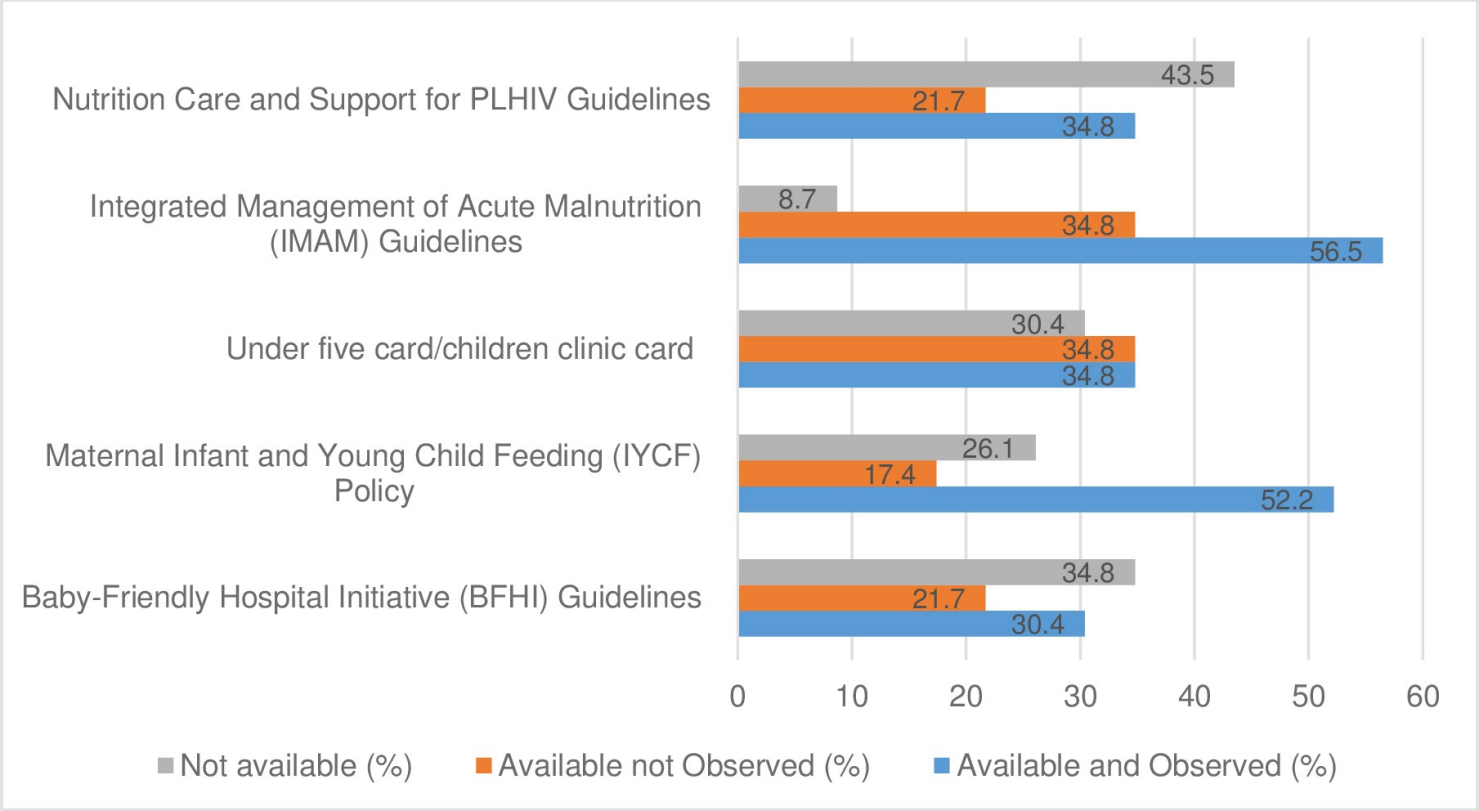
Availability of protocols and guidelines (n = 23).

Researchers verified the presence of protocols and guidelines by asking the respondents to show them copies of the documents. The majority of respondents reported having the Integrated Management of Acute Malnutrition and the Maternal Infant and Young Child Feeding Guidelines (56.5% and 52.2% respectively) (Fig 1). All respondents who reported that their facilities had a Maternal Infant and Young Child Feeding Policy, under-five card/children clinic cards and Integrated Management of Acute Malnutrition guidelines also reported having implemented these documents (Table 4). However, a number of key guidelines and documents were lacking. Only 34.8% of the respondents reported having under-five cards/children clinic cards that were verified by researchers. Additionally, 43.5% and 34.8% of the respondents reported not having nutrition care and support for People Living with HIV (PLHIV) guidelines and baby-friendly hospital initiative guidelines respectively.

**Table 4:** Protocols and guidelines used/implemented.

Protocols and guidelines Used/Implemented	Implemented/used n(%)	Not implemented/used n(%)
Baby-Friendly Hospital Initiative (BFHI) Guidelines (N = 12)	9(75)	3(25)
Maternal Infant and Young Child Feeding (IYCF) Policy (N = 16)	16(100)	0
Under five card/children clinic card (N = 16)	16(100)	0
Integrated Management of Acute Malnutrition (IMAM) Guidelines (N = 21)	21(100)	0
Nutrition Care and Support for PLHIV Guidelines (N = 13)	12(92.3)	1(7.7)

### Human resource

Table 5 outlines the available human resources at the sampled hospitals by Province. Hospitals in Luapula and Muchinga Provinces had an average of two Medical Officers per hospital who also provided nutritional services. However, a level-one hospital in Muchinga Province reported that they lacked Medical Officers. Level-one hospitals in Western province had the lowest average number of nurses/midwives per hospital with only about eight nurses/midwives per hospital. Almost all the hospitals sampled had an average of one to two Nutritionists in each hospital with all of them providing nutritional services, however, Luapula, Northern and North-Western provinces had a hospital that reported not having a nutritionist. Hospitals in Northern Province had the highest average number of Health Educators with about seven Health Educators per facility while Western Province had the lowest, averaging about one per hospital.

**Table 5:** Distribution of human resource by province.

Indicator by Province	Luapula N = 5	Muchinga N = 4	Northern N = 5	North-Western N = 6	Western N = 5
	Mean(SD)	Mean(SD)	Mean(SD)	Mean(SD)	Mean(SD)
**Medical Officer/Doctors**					
Average number of Medical Officers/Doctors assigned to hospitals	2.4 (0.9)	2.3 (1.7)	1.6 (0.5)	1.5 (0.8)	1.6 (0.5)
Number of hospitals with no Medical Officers/Doctors	0	1	0	1	0
Average # of Medical Officers/Doctors who usually provide nutrition services	2.4 (0.9)	1.8 (2.1)	0.8 (0.8)	1 (0.9)	1.2 (0.8)
Average # of Medical Officers/Doctors received nutrition training past 3 years	1.6 (1.5)	1.5 (1.9)	0.6 (0.8)	0.8 (0.9)	0.2 (0.4)
**Nurse/midwife**					
Average number of Nurse/Midwife assigned to hospitals	34.2 (28)*	9.8 (1.7)	19.4 (10.5)	23 (19.9)	8.4 (1.3)
Number of hospitals with no Nurse/Midwife	0	0	0	1	0
Average # of Nurse/Midwife who usually provide nutrition services	7.4 (3.5)	0	0.2 (0.4)	0.8 (1.3)	6.4 (6.1)
Average # of Nurse/Midwife received nutrition training past 3 years	3.6 (4.9)	0	0.8 (1.3)	0	0.2 (0.4)
**Nutritionist**					
Average number of Nutritionist assigned to hospitals	1 (0.7)	1.5 (0.6)	0.8 (0.4)	1.2 (1)	1.8 (0.7)
Number of hospitals with no Nutritionist	1	0	1	1	0
Average # of Nutritionist who usually provide nutrition services	1 (0.7)	1.5 (0.6)	0.8 (0.4)	1.2 (1)	1.8 (0.8)
Average # of Nutritionist received nutrition training past 3 years	1 (0.7)	1.5 (0.6)	0.8 (0.4)	0.8 (0.4)	1.6 (0.9)
**Health educator**					
Average number of Health Educator assigned to hospitals	5.6 (3.3)	2.5 (4.4)	7.4 (6.5)	5.8 (5.5)	0.8 (0.8)
Number of hospitals with no Health Educators	0	2	1	1	2
Average # of Health Educators who usually provide nutrition services	5 (3)	2.5 (4.4)	3 (3.7)	2.2 (4.8)	0.6 (0.9)
Average # of Health Educators received nutrition training past 3 years	2 (2)	2.5 (4.4)	1.6 (3)	0	0

### Supervision, mentorship and quality improvement

**Fig 2:**
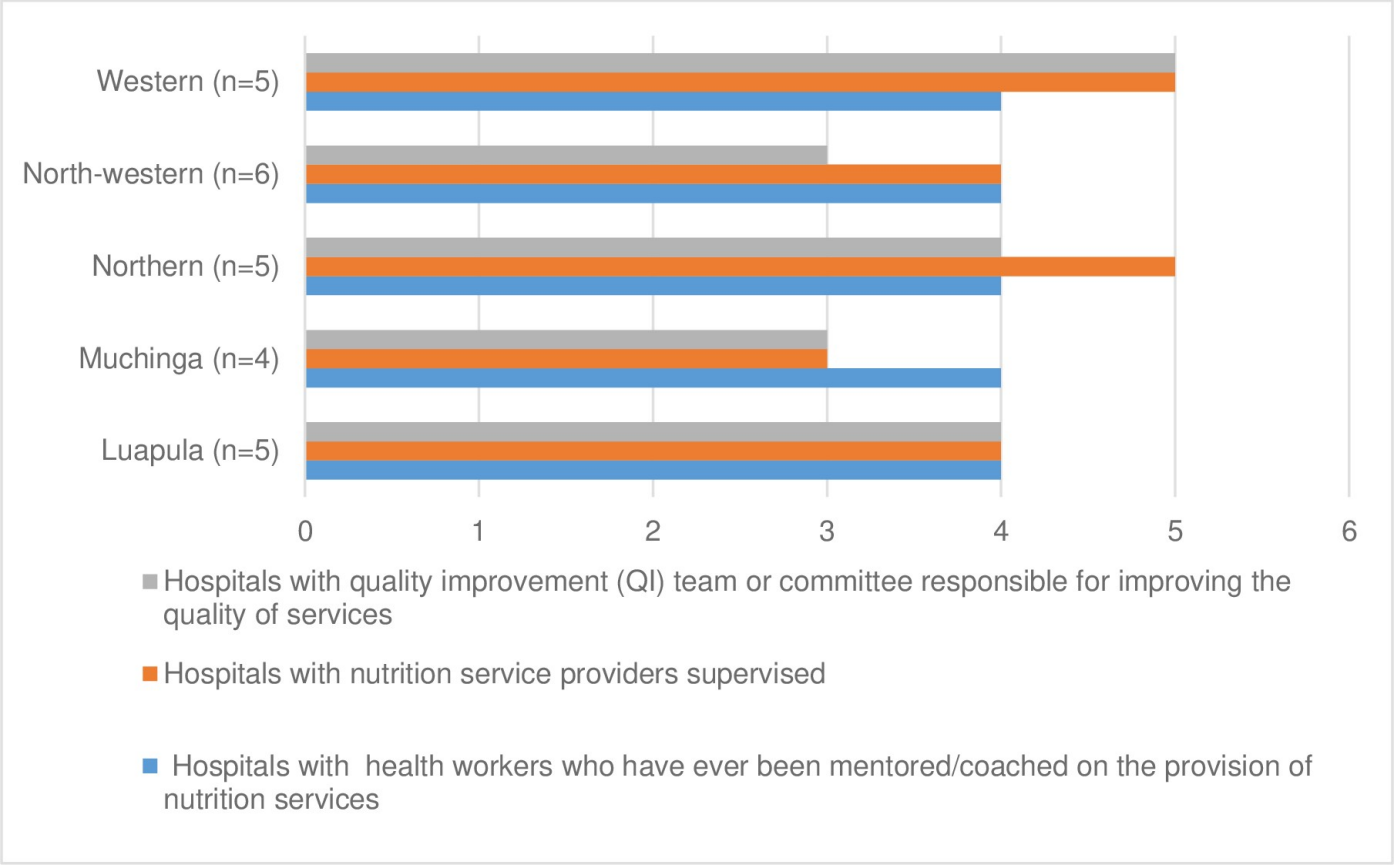
Supervision, mentorship and quality improvement.

Fig 2 shows that all of the sampled hospitals in Western Province reported having a quality improvement committee and providing supervision to staff who provided nutritional services. Four out of six hospitals in North-Western Province reported providing supervision and mentorship to staff who provided nutritional services. Additionally, three out of the six hospitals in North-Western Province had a quality improvement team set up.

### Availability and stock-outs of nutritional drugs and supplements

**Fig 3:**
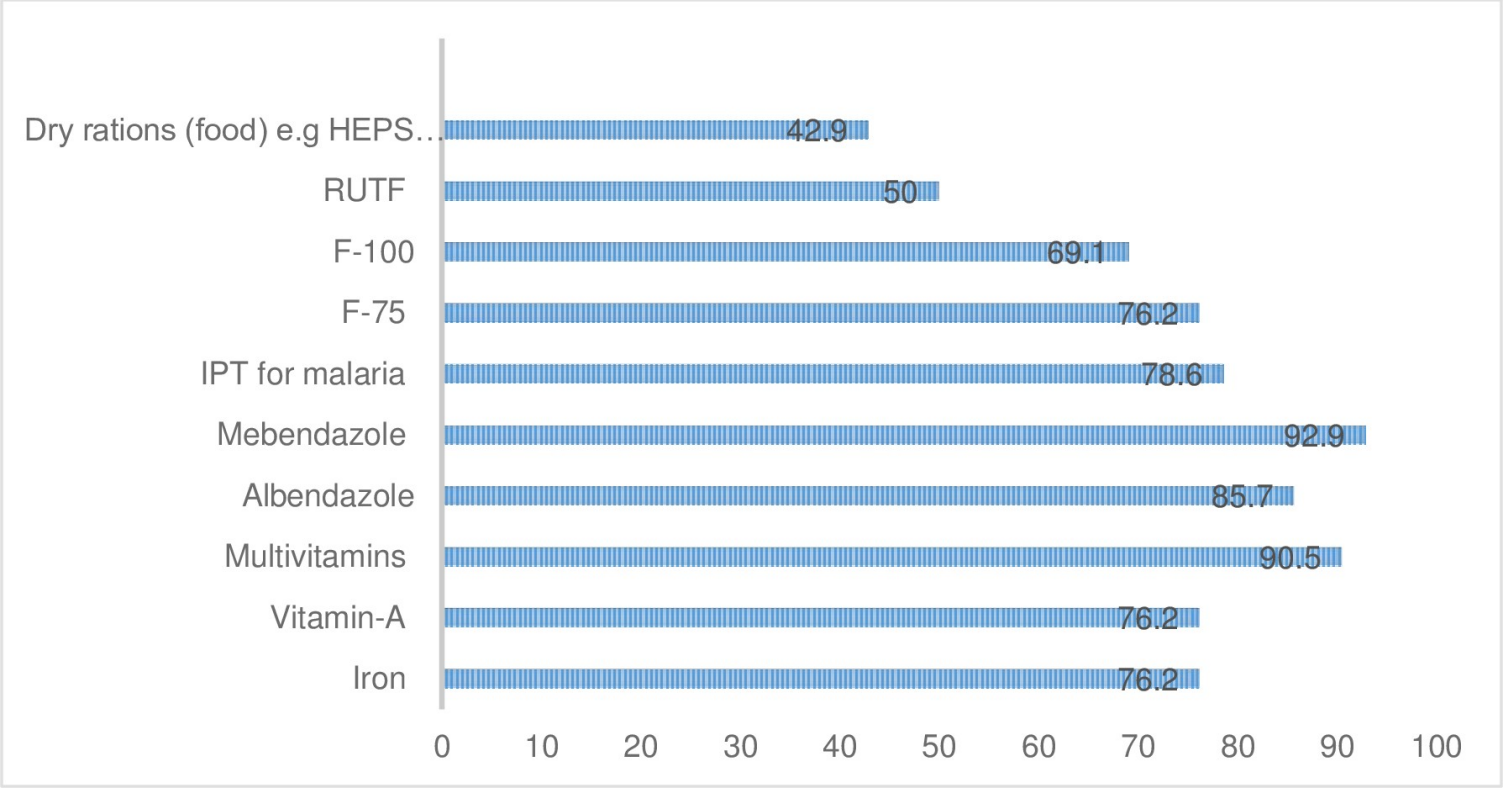
Drugs managed at hospital (n = 42).

The majority of the hospitals reported using folic acid, mebendazole, and multivitamins (95.2%, 92.9% and 90.5%, respectively) to manage cases of malnutrition. However, only 42.9% used dry rations (e.g HEPS Combined Mineral Vitamin) to treat cases of malnutrition (Fig 3).

About 53% of the respondents reported having stock-outs of multivitamins at the time of the study. The majority of the participants also reported having stock-outs of F-75, F-100 and Ready to Use Therapeutic Food (RUTF) (65.6%, 65.5% and 71.4%, respectively) at the time of the study (Table 6).

**Table 6:** Stock-outs at time of survey.

Drug	Stock-out (n%)	No stock-out (n%)	Don’t Know (n%)
Folic Acid (n = 40)	6(15)	30(75)	4(10)
Iron (n = 32)	4(12.5)	24(75)	4(12.5)
Vitamin-A (n = 32)	10(31.3)	19(59.4)	3(9.4)
Multivitamins (n = 38)	20(52.6)	13(34.2)	5(13.2)
Albendazole (n = 36)	10(27.8)	21(58.3)	5(13.9)
Mebendazole (n = 39)	14(35.9)	21(53.9)	4(10.3)
IPT for malaria (n = 33)	8(24.2)	22(66.7)	3(9.1)
F-75 (n = 32)	21(65.6)	9(28.1)	2(6.3)
F-100 (n = 29)	19(65.5)	7(24.1)	3(10.3)
RUTF (n = 21)	15(71.4)	4(19.1)	2(9.5)
Dry rations (food) e.g HEPS Combined Mineral Vitamin (n = 19)	7(38.9)	8(44.4)	3(16.7)

## Discussion

This study was designed to analyse and describe the health system capacities that exist and the services provided by first level hospitals in the five ZHSIP Provinces of Zambia in order to identify gaps and recommend actions for Zambia to achieve universal coverage of essential nutrition services at this level of primary healthcare. The essential nutrition services assessed were: nutrition counseling (group and individual), engagement of CBVs as part of the nutrition care system, CBVs providing nutrition services, referral of clients to hospitals by CBVs or other social services providers, counter-referral of clients by hospitals to CBVs or other social services providers. Our results show that systems for referrals from the community to the hospitals and counter-referrals from hospitals to the community are a weak link in the care continuum for malnourished children in this study population as can be seen from the fact that only 48% of hospitals reported having a system of referral of malnourished children to the community and only 56% of community based volunteers reported having a system of referral to hospitals. It is important to note that both the CBVs and health workers at health facilities are critical to the management of SAM and a well-functioning bi-directional referral system is vital. A joint statement by the World Health Organization (WHO), World Food Program (WFP), and United Nations System Standing Committee on Nutrition, and United Nations Children’s Fund (UNICEF), recognises the value of community-based management of SAM and says “if properly combined with a facility-based approach for those malnourished children with medical complications and implemented on a large scale, community-based management of SAM could prevent the deaths of hundreds of thousands of children” [6]. The same statement advises to save children’s lives by adopting policies and programs that ‘establish adequate referral arrangements for children suffering from complicated forms of severe acute malnutrition.’ Therefore, ideally all the first level hospitals sampled should have systems for referrals between them and the community.

In addition to the need to have a functioning referral system, management of SAM requires constant supplies of therapeutic feeds. Outpatient therapeutic feeding in Zambia involves assessing and observing each child’s feeding to determine if there is a problem. Treating and managing SAM children without medical complications includes providing RUTF to families of children [7–11]. However, our findings show that about 71% of the respondents reported having stock outs of RUTF at the time of the study. This poses a big challenge in the management or prevention of SAM. What our study was unable to elucidate are the reasons why there were shortages of RUTFs; the possibility may be that these are all imported and hence more expensive to sustain the supplies. Zambia will need to explore the possibility of developing locally manufactured therapeutic feeds using local foods. Inpatient care for cases of malnutrition involves provision of a broad spectrum of antibiotics upon admission. If no response is observed, next line of treatment is administered as necessary. Other necessary treatments include administering of Vitamin A, liquid feeding, feeding children who are able to tolerate solids for 4–5 weeks, and commencement of nutritional therapeutic management of SAM when a child is ready, and use of several monitoring charts to assess response to therapeutic feeds. However, our findings show that about 31% of the respondents reported having stock out of Vitamin A at the time of the study, additionally, a study conducted in 2015[12] to investigate the capacity of rural health workers in treating acute malnutrition in Southern Province of Zambia revealed that there were inadequate resources for health workers to manage the high rate of acute malnutrition.

### Availability of protocol guidelines

Protocols and guidelines have been shown to reduce patient harm as well as improve health care through standardization and communication [13–19]. The American College of Obstetricians and Gynecologists [20] records that implementation of protocols and guidelines often is delayed because of lack of health care provider awareness or difficult clinical algorithms in medical institutions. However, the use of checklists and protocols clearly has been demonstrated to improve outcomes and their use is strongly encouraged. Their use in any working environment including those providing health care such as nutrition services cannot be over emphasized. This study assessed the presence of various protocols and guidelines that are important in the provision of quality nutritional services in the visited facilities; and where the guidelines were available, the study assessed the extent to which they were utilized. Results show that close to half (43.5%) of the facilities (n = 23) had no guidelines on nutrition care and support for People Living with HIV (PLHIV) guidelines. With high prevalence of HIV/AIDS in Zambia [21] lack of guidelines for PLHIV in most facilities does not only limit the services infected children receive, but also the quality provided.

However, it is important to note that the results show that 91.3% of the hospitals visited (n = 23) had guidelines on Integrated Management of Acute Malnutrition (IMAM). This is an important finding because it implies that if health workers are sensitized enough to use these guidelines and they do actually use them, the management of children with SAM is likely to improve. Sensitization is important because the results of this study show that most of the recommended guidelines were not utilized in most of the sampled facilities.

Other guidelines and protocols that were available in the hospitals include Baby-Friendly Hospital Initiative (BFHI) Guidelines (52.1), maternal Infant and Young Child Feeding (IYCF) Policy (69.6%), under five card/children clinic card (69.6%). Therefore, most of the hospitals are expected to provide standardized and quality services with regards to nutrition as more than half of the surveyed ones had all the guidelines required in the provision of nutrition services for children.

### Availability of equipment at facilities

The results show that different equipment and tools for provision of nutrition services were available at most the sampled health facilities. Close to three-quarters (73.9%) of the hospitals had infant/pediatric scales in the nutrition department and more than half (50%) of them had at least two functional scales in the hospital. However, the study established that a number of equipment were missing in the facilities and even for those available, a number of them were not functional. This is an important gap to bridge as lack of these equipment can prevent better nutrition assessment as established by a study by Billah [22] which concluded that an overall lack of essential equipment including scales, stadiometers and growth monitoring cards prevent appropriate nutritional assessment and screening for under nutrition.

### Human resource availability

Availability of human resource is critical in reduction of malnutrition and promoting a healthy lifestyle among Paediatrics and adults. According to the Ministry of Health structure, a level one hospital is expected to have four Medical officers/Doctors, eighty-one Nurses/Midwives and two nutritionists. However, a description of the available human resources at the sampled hospitals by Province shows averages of about two medical officers/doctors per hospital. The results also show the highest average number of nurses in Luapula province of about thirty-four nurses, whereas, Western province had the lowest average of about eight nurses per hospital. On the other hand, despite some hospitals not having nutritionists, the average number of nutritionists in most of the hospitals in the Provinces ranged between one and two. This discrepancy in the human resource between what was occurring on the ground versus the established structure by the Ministry of Health as at 2018 could have contributed to limiting the overall outcomes of the efforts being implemented in targeting factors that cause malnutrition, as lack of professional advice from the hospital denies the clients the much needed information to improve their nutrition status. The uneven distribution of health staff in all provinces could also have affected the overall performance of programs targeted on healthy nutrition and malnutrition in various populations.

## Conclusion

This study has revealed a number of gaps in the health system and health service delivery that requires to be addressed; most notably, a lack of tools, policies and guidelines, drugs and health specialists to help care for malnourished infants and children. Our findings also reveal inadequate referral systems between the community and health facilities in the management of severe acute malnutrition. Achieving universal coverage for nutrition services in Zambia will require a lot more attention to the health systems issues found in this study.

### Limitation

We recognize some limitations of the study. We sampled up to six hospitals in each Province without calculating a statistically significant sample and this may pose potential risk of bias to the measures. We also purposively sampled the key informants interviewed in the facility assessment tool based on their designation in the hospital as the tool required which may not give estimates that represents what exists in the entire Country. Notwithstanding this limitation, we felt that the study provides important findings as the facilities were randomly selected and these results can guide deliberate strategies to intensify the current efforts to address malnutrition among children under five.

## Supporting information

S1 Dataset (DTA)

S1 Questionnaire Facility assessment questionaire; module 1 and 4 of the spring tool. (DOCX)
