# Supplementary material for: Towards universal coverage for nutrition services in children under five years—A descriptive analysis of the capacity of level one hospitals to provide nutrition services in five provinces of Zambia
Source: PLoS One. 2020 May 12;15(5):e0232663. doi: 10.1371/journal.pone.0232663 (PMC7217438; doi:10.1371/journal.pone.0232663)
Supplement: S1 Questionnaire — (DOCX) [file pone.0232663.s002.docx]

FACILITY ASSESSMENT QUESTIONAIRE; MODULE 1 AND 4 OF THE SPRING TOOL

| **MODULE 1. INTERVIEW WITH THE HEALTH FACILITY MANAGER** | | | | | | | | | | | | |
| --- | --- | --- | --- | --- | --- | --- | --- | --- | --- | --- | --- | --- |
| THE INFORMATION IN Q001-007 SHOULD BE PRE-FILLED BY THE DATA COLLECTION TEAM PRIOR TO THE INTERVIEW.  IF THIS TOOL IS BEING USED FOR THE ENTIRE FACILITY, ENTER ‘99’ FOR THE UNIT CODE (Q002). | | | | | | | | | | | | |
| **FACILITY INFORMATION** | | | | | | | | | | | | |
| \| … \|  \| \| --- \| --- \|   001 NAME OF FACILITY___________________________ FACILITY CODE………………………  002 UNIT CODE………………………………………………………………………………………………………………………  003 DISTRICT___________________________________ DISTRICT CODE………………………..   \|  \|  \| \| --- \| --- \|   004 SUB-COUNTY_______________________________ SUB-COUNTY CODE…………………   \| … \|  \| \| --- \| --- \|   005 TYPE OF FACILITY____________________________ TYPE OF FACILITY CODE………… | | | | | | | | | | | | |
| **INTERVIEW INFORMATION** | | | | | | | | | | | | |
|  | | | | | | | | | | | | |
| 006 DATE………………..……………………………………….Day  007 INTERVIEWER NAME________________________ | |  | |  | Month Year  INTERVIEWER CODE……………….……… | |  |  |  |  | |  |
|  |  |  | | |  |  |  | | | | |  |
| **Consent** | | | | | | | | | | | | |
| 008 | RECORD WHETHER PERMISSION WAS RECEIVED FROM THE RESPONDENT. | | | | | YES ....................................................... 1  NO ........................................................ 2 | | | | | 🡪END | |
| **HEALTH FACILITY CHARACTERISTICS** | | | | | | | | | | | | |
| I would like to begin the interview by asking you about the overall facility organization and availability of services. | | | | | | | | | | | | |
| 101 | Who is the managing authority of this health facility? | | Government ............................................................................... 1  NGO .............................................................................................. 2  Faith-based (NGO) .................................................................. 3  Private for profit ....................................................................... 4  Private not for profit ............................................................... 5  Other (SPECIFY) ........................................................................ 6 | | | | | | | | | |
| 102 | What is the estimated population to be covered by the facility? In other words, what is the catchment population?  IF THE RESPONDENT DOES NOT KNOW,  RECORD ‘8888888’ IN THE BOXES PROVIDED. | | \|  \|  \|  \|  \|  \|  \|  \| \| --- \| --- \| --- \| --- \| --- \| --- \| --- \|   POPULATION ........................................ | | | | | | | | | |

| **NUTRITION SERVICES** | | | |
| --- | --- | --- | --- |
| Now I am interested in knowing more about the nutrition services provided in this facility. | | | |
| 103 | Does this facility provide group counseling or education on nutrition?  IF YES, ASK: Could you describe the format and content of the group counseling or education provided?  IF NO, ASK: Why not? | YES .......................................... 1  NO .......................................... 2  DON’T KNOW .................... 8 |  |
| 104 | Does this facility counsel clients individually on nutrition?  IF YES, ASK: Could you describe the format and content of the individual counseling provided?  IF NO, ASK: Why not? | YES .......................................... 1  NO .......................................... 2  DON’T KNOW .................... 8 |  |
| 105 | Does this facility engage community workers/volunteers in and/or outside facilities as part of the care system?  IF YES, ASK: How?  IF NO, ASK: Why not? | YES .......................................... 1  NO .......................................... 2  DON’T KNOW .................... 8 | 🡪109  🡪109 |
| 106 | Do the community workers/volunteers provide nutrition services?  IF YES, ASK: Could you describe the services provided?  IF NO, ASK: Why not? | YES .......................................... 1  NO .......................................... 2  DON’T KNOW .................... 8 |  |
| 107 | Do community workers/volunteers or other government or NGO social services refer clients to this facility?  IF YES, ASK: When or why?  IF NO, ASK: Why not? | YES .......................................... 1  NO .......................................... 2  DON’T KNOW .................... 8 |  |
|  |  |  |  |
| 108 | Does this facility have a system for referring clients to any other facility and/or community-based services? Communitybased support services might include:   - Economic and livelihoods development (e.g. business development, vocational training, etc.) - Food security support - HIV counseling and testing - Home care - PLHIV client treatment support (e.g. reminding to take ARVs, following up with clients using therapeutic foods) - Social support for PLHIV - Community based nutrition screening/monitoring - Social support for mothers (e.g. breastfeeding support groups)   These services may be provided by government or nongovernmental programs, projects, or organizations.  IF YES, ASK: Could you please explain the system?  IF NO, ASK: Why not? | YES .......................................... 1  NO .......................................... 2  DON’T KNOW .................... 8 |  |

| **EQUIPMENT AND TOOLS FOR ASSESSMENT OF NUTRITION STATUS** | | | |
| --- | --- | --- | --- |
| Now I would like to ask you questions about the equipment and tools available in your facility for providing nutrition services. | | | |
| 109_1 | Is an **infant/pediatric scale** available in your facility?  If YES, ASK: Is it assigned to this facility or shared with another facility? | YES, ASSIGNED ...................1  YES, SHARED ........................2  NO ...........................................3  N/A ..........................................9 | - 110_1 - 110_1 |
| 109_2 | How many are in working condition?  RECORD THE NUMBER IN THE SPACE PROVIDED. IF THE  RESPONSE IS LESS THAN 10, YOU SHOULD FILL IN LEADING  ZEROES. FOR EXAMPLE, A RESPONSE OF ‘7’ SHOULD BE  RECORDED ‘07’ IN TWO BOXES. RECORD ‘88’ IF NOT KNOWN. | \|  \|  \| \| --- \| --- \| |  |

| 109_3 | RECORD THE DAY, MONTH, AND YEAR OF THE LAST CALIBRATION IF UNKNOWN, RECORD ‘88’ OR ‘8888’ IN THE BOXES PROVIDED. | \|  \|  \| \| --- \| --- \|   DAY ..................................   \|  \|  \| \| --- \| --- \|   MONTH..........................  YEAR ................. |  |
| --- | --- | --- | --- | --- | --- | --- | --- |
| 110_1 | Is an **adult weighing scale** available in your facility?  IF YES, ASK: Is it assigned to this facility or shared with another facility? | YES, ASSIGNED .................. 1  YES, SHARED ....................... 2  NO .......................................... 3  N/A ......................................... 9 | - 111_1 - 111_1 |
| 110_2 | How many are in working condition?  RECORD THE NUMBER IN THE SPACE PROVIDED. FILL IN  LEADING ZEROES AS NECESSARY. RECORD ‘88’ IF NOT KNOWN. | \|  \|  \| \| --- \| --- \| |  |
| 110_3 | RECORD THE DAY, MONTH, AND YEAR OF THE LAST CALIBRATION IF UNKNOWN, RECORD ‘88’ OR ‘8888’ IN THE BOXES PROVIDED. | \|  \|  \| \| --- \| --- \|   DAY ..................................   \|  \|  \| \| --- \| --- \|   MONTH..........................   \|  \|  \|  \|  \| \| --- \| --- \| --- \| --- \|   YEAR ............... |  |
| 111_1 | Is a **length board** available in your facility?  IF YES, ASK: Is it assigned to this facility or shared with another facility? | YES, ASSIGNED .................. 1  YES, SHARED ....................... 2  NO .......................................... 3  N/A ......................................... 9 | 🡪112_1  🡪112_1 |
| 111_2 | How many are available?  RECORD THE NUMBER IN THE SPACE PROVIDED. FILL IN LEADING ZEROES AS NECESSARY. RECORD ‘88’ IF NOT KNOWN. | \|  \|  \| \| --- \| --- \| |  |
| 112_1 | Is a **stadiometer (for adults)** available in your facility?  IF YES, ASK: Is it assigned to this facility or shared with another facility? | YES, ASSIGNED .................. 1  YES, SHARED ....................... 2  NO .......................................... 3  N/A ......................................... 9 | - 113_1 - 113_1 |
| 112_2 | How many are in working condition??  RECORD THE NUMBER IN THE SPACE PROVIDED. FILL IN  LEADING ZEROES AS NECESSARY. RECORD ‘88’ IF NOT KNOWN. | \|  \|  \| \| --- \| --- \| |  |
| 113_1 | Is a **MUAC tape for children** available in your facility?  IF YES, ASK: Is it assigned to this facility or shared with another facility? | YES, ASSIGNED .................. 1  YES, SHARED ....................... 2  NO .......................................... 0 N/A ......................................... 9 | - 114_1 - 114_1 |
| 113_2 | How many are available?  RECORD THE NUMBER IN THE SPACE PROVIDED. FILL IN  LEADING ZEROES AS NECESSARY. RECORD ‘88’ IF NOT KNOWN. | \|  \|  \|  \| \| --- \| --- \| --- \| |  |
| 114_1 | Is a **MUAC tape for adults** available in your facility?  IF YES, ASK: Is it assigned to this facility or shared with another facility? | YES, ASSIGNED .................. 1  YES, SHARED ....................... 2  NO .......................................... 3  N/A ......................................... 8 | - 115_1 - 115_1 |
| 114_2 | How many are available?  RECORD THE NUMBER IN THE SPACE PROVIDED. FILL IN LEADING ZEROES AS NECESSARY. RECORD ‘888’ IF NOT KNOWN. | \|  \|  \|  \| \| --- \| --- \| --- \| |  |
| 115_1 | Is a **MUAC tape for pregnant and lactating women** available in your facility?  IF YES, ASK: Is it assigned to this facility or shared with another facility? | YES, ASSIGNED .................. 1  YES, SHARED ....................... 2  NO .......................................... 3  N/A ......................................... 8 | - 116_1 - 116_1 |
| 115_2 | How many are available?  RECORD THE NUMBER IN THE SPACE PROVIDED. FILL IN LEADING ZEROES AS NECESSARY. RECORD ‘888’ IF NOT KNOWN. | \|  \|  \|  \| \| --- \| --- \| --- \| |  |

| **PROTOCOLS AND IEC MATERIALS** | | | |
| --- | --- | --- | --- |
| Now I am going to ask you about various national guidelines, protocols, and flyers available at this facility. I would like to see as many of these as I can. | | | |
| READ THE NAME OF EACH GUIDELINE/PROTOCOL LISTED. RECORD WHETHER THE SPECIFIC VERSION OF THE  GUIDELINE/ PROTOCOL IS OBSERVED, REPORTED BUT NOT SEEN, NOT AVAILABLE, OR IF THE RESPONDENT DOES NOT KNOW. IF YES, OBSERVED OR NOT, ASK THE RESPONDNET IF IT BEING USED. IF YES, ASK HOW AND IF NOT, ASK WHY NOT.  [NOTE: FOR EACH DOCUMENT IT WILL BE IMPORTANT TO DETERMINE THE YEAR OF THE MOST RECENT  VERSION OR THE VERSION THAT FACILITIES ARE EXPECTED TO HAVE.] | | | |
| 116_1 | Do you have the [DATE] **Baby-Friendly Hospital Initiative (BFHI) Guidelines**?    IF YES, ASK: Can I see a copy of it? | YES, OBSERVED .................. 1 YES, NOT OBSERVED ....... 2  NO ........................................... 3  DON’T KNOW ..................... 8 | - 117_1 - 117_1 |

| 116_2 | Is it being implemented or used in this facility? | | YES .......................................... 1  NO ........................................... 2  DON’T KNOW ..................... 8 |  |
| --- | --- | --- | --- | --- |
| 116_3 | IF YES, ASK: Could you explain how? IF NO, ASK:  Could you explain why not? |  |  |  |
| 117_1 | Do you have the [DATE] **Infant and Young Child Feeding (IYCF) Policy**?    IF YES, ASK: Can I see a copy of it? | | YES, OBSERVED .................. 1 YES, NOT OBSERVED ....... 2  NO ........................................... 3  DON’T KNOW ..................... 8 | - 118_1 - 118_1 |
| 117_2 | Is it being implemented or used in this facility? | | YES .......................................... 1  NO ........................................... 2  DON’T KNOW ..................... 8 |  |
| 117_3 | IF YES, ASK: Could you explain how? IF NO, ASK:  Could you explain why not? |  |  |  |
| 118_1 | Do you have [DATE] **child health cards**?    IF YES, ASK: Can I see a copy of it? | | YES, OBSERVED .................. 1 YES, NOT OBSERVED ....... 2  NO ........................................... 3  DON’T KNOW ..................... 8 | - 119_1 - 119_1 |
| 118_2 | Are they being used in this facility? | | YES .......................................... 1  NO ........................................... 2  DON’T KNOW ..................... 8 |  |
| 118_3 | IF YES, ASK: Could you explain how? IF NO, ASK:  Could you explain why not? |  |  |  |
| 119_1 | Do you have the [DATE] **Integrated Management of Acute Malnutrition (IMAM) Guidelines**?    IF YES, ASK: Can I see a copy of it? | | YES, OBSERVED .................. 1 YES, NOT OBSERVED ....... 2  NO ........................................... 3  DON’T KNOW ..................... 8 | - 120_1 - 120_1 |
| 119_2 | Is it being implemented or used in this facility? | | YES .......................................... 1  NO ........................................... 2  DON’T KNOW ..................... 8 |  |

| 119_3 | IF YES, ASK: Could you explain how? IF NO, ASK:  Could you explain why not? |  |  |  |
| --- | --- | --- | --- | --- |
| 120_1 | Do you have the [DATE] **Nutrition Care and Support for PLHIV Guidelines**?    IF YES, ASK: Can I see a copy of it? | | YES, OBSERVED .................. 1 YES, NOT OBSERVED ....... 2  NO ........................................... 3  DON’T KNOW ..................... 8 | - 121 - 121 |
| 120_2 | Is it being implemented or used in this facility? | | YES .......................................... 1  NO ........................................... 2  DON’T KNOW ..................... 8 |  |
| 120_3 | IF YES, ASK: Could you explain how? IF NO, ASK:  Could you explain why not? |  |  |  |
| 121 | Are there any other tools and/or guidelines being implemented or used for nutrition services available in this facility?  Can I see a copy of these? | | YES, OBSERVED .................. 1 YES, NOT OBSERVED ....... 2  NO, ........................................ 3  DON’T KNOW ..................... 8 | - 201 - 201 |
| 122_1 | RECORD TITLE:       \|  \|  \|  \|  \| \| --- \| --- \| --- \| --- \|   RECORD YEAR:  IF YEAR IS UNKNOWN, RECORD ‘9999’ IN THE SPACE PROVIDED. | | YES, OBSERVED .................. 1 YES, NOT OBSERVED ....... 2  NO, .......................................... 3  DON’T KNOW ..................... 8 |  |
| 122_2 | IF YES, ASK: Could you explain how? IF NO, ASK:  Could you explain why not? |  |  |  |
| 123_1 | RECORD TITLE:       \|  \|  \|  \|  \| \| --- \| --- \| --- \| --- \|   RECORD YEAR:  IF YEAR IS UNKNOWN, RECORD ‘9999’ IN THE SPACE PROVIDED. | | YES, OBSERVED .................. 1 YES, NOT OBSERVED ....... 2  NO, .......................................... 3  DON’T KNOW ..................... 8 |  |
| 123_2 | IF YES, ASK: Could you explain how? IF NO, ASK:  Could you explain why not? |  |  | |
| 124_1 | RECORD TITLE:       \|  \|  \|  \|  \| \| --- \| --- \| --- \| --- \|   RECORD YEAR:  IF YEAR IS UNKNOWN, RECORD ‘9999’ IN THE SPACE PROVIDED. | | OBSERVED ........................... 1  NOT OBSERVED ................. 2 |  |
| 124_2 | IF YES, ASK: Could you explain how? IF NO, ASK:  Could you explain why not? |  |  | |

| **HUMAN RESOURCES** | | | | | | | | | | |
| --- | --- | --- | --- | --- | --- | --- | --- | --- | --- | --- |
| Now I am interested in asking you questions about human resources and human resource management. | | | | | | | | | | |
| 125 | First, I would like to ask you about the number of each type/cadre of health worker at this facility. I am only interested in health workers who are employed by this health facility. This does not include volunteers or seconded staff.   1. READ THE PROVIDER TYPE (CADRE), THEN ASK: How many [PROVIDER TYPE] are assigned to the [UNIT NAME] unit?   RECORD THE NUMBER OF PROVIDERS IN THE SPACE PROVIDED. IF THE RESPONDENT DOES NOT KNOW, RECORD ‘99’.   1. NEXT ASK: Does [PROVIDER TYPE] usually provide nutrition services in this facility? EXPLAIN: By nutrition services, I mean assessment of nutritional status, nutrition counseling, nutrition support, and/or referral to nutrition support services.   RECORD THE NUMBER OF PROVIDERS THAT USUALLY PROVIDE NUTRITION SERVICES IN THE SPACE PROVIDED. IF THE RESPONDENT DOES NOT KNOW, RECORD ‘99’.   1. FINALLY, ASK: Has [PROVIDER TYPE] been trained to provide nutrition services in this facility? EXPLAIN: By training, I mean pre-service or in-service training.   RECORD THE NUMBER OF PROVIDERS THAT HAVE BEEN TRAINED IN PROVIDING NUTRITION SERVICES IN THE SPACE PROVIDED. IF THE RESPONDENT DOES NOT KNOW, RECORD ‘99’.  Note: Revise provider types/cadres and units as well as the type of training as appropriate for the country context and evaluation objectives. | | | | | | | | | |
| **Provider Type / Cadre** | | **A. How many**  **[PROVIDER TYPE] are assigned to the**  **[UNIT NAME] unit?** | | | **B. How many**  **[PROVIDER**  **TYPE] usually provide nutrition services in the**  **[UNIT NAME] unit?** | | | **C. How many**  **[PROVIDER TYPE] have been trained in the past three years to provide nutrition services in the**  **[UNIT NAME] unit?** | | |
|  |  | **[UNIT NAME]** | | | **[UNIT NAME]** | | | **[UNIT NAME]** | | |
|  |  | (  a) ANC | (  c) PEDIATRIC | (  d) HIV/TB | (  a) ANC | (  c) PEDIATRIC | (  d) HIV/TB | (  a) ANC | (  c) PEDIATRIC | (  d) HIV/TB |
| 125_1 | PEDIATRICIAN |  |  |  |  |  |  |  |  |  |
| 125_2 | OB/GYN |  |  |  |  |  |  |  |  |  |
| 125_3 | GENERAL PHYSICIAN |  |  |  |  |  |  |  |  |  |
| 125_4 | MEDICAL OFFICER |  |  |  |  |  |  |  |  |  |
| 125_5 | NURSE |  |  |  |  |  |  |  |  |  |
| 125_6 | MIDWIFE |  |  |  |  |  |  |  |  |  |
| 125_7 | NURSING ASSISTANT, AID,  AUXILLIARY |  |  |  |  |  |  |  |  |  |
| 125_8 | NUTRITIONIST |  |  |  |  |  |  |  |  |  |
| 125_9 | HEALTH EDUCATOR / SOCIAL  WORKER / COUNSELOR |  |  |  |  |  |  |  |  |  |

COMMENTS ON WHY CERTAIN CADRES DO NOT PROVIDE NUTRITION SERVICES:

`

| **MENTORING / COACHING** | | | |
| --- | --- | --- | --- |
| Now I would like to ask you about mentoring or coaching conducted in this facility. | | | |
| 126 | Are health workers ever mentored/coached on the provision of nutrition services at this facility? | YES ...............................................1  NO ................................................2  DON’T KNOW ......................... 8 |  |
|  | IF YES, ASK: Could you describe the mentoring/coaching provided? IF NO, ASK: Why not? | | |
|  |  | | |
|  |  | | |
|  |  | | |

| **SUPERVISION & FEEDBACK** | | | |
| --- | --- | --- | --- |
| Next, I would like to ask you about any supervision of health providers that is conducted in this facility. | | | |
| 127 | Are nutrition service providers in this facility ever supervised? | YES ............................................... 1  NO................................................ 2  DON’T KNOW......................... 8 | 🡪130  🡪130 |
|  | IF YES, ASK: Could you describe the supervisory visits? IF NO, ASK: Why not? | | |
|  |  | | |
|  |  | | |
|  |  | | |
| 128 | How many times in the past year have nutrition service providers in this facility been supervised? | NEVER ......................................... 0  ONCE .......................................... 1  2-3 TIMES .................................. 2  4-5 TIMES .................................. 3  ≥ 6 TIMES .................................. 4  DON’T KNOW.......................... 8 |  |
| 129 | Is feedback (either positive or negative) provided to nutrition service providers based on the supervision? | YES ............................................... 1  NO................................................ 2  DON’T KNOW.......................... 8 |  |

| **QUALITY IMPROVEMENT** | | | |
| --- | --- | --- | --- |
| Now I would like to understand any systems in place to improve the quality of care in this facility. This will involve asking to see records and documentation. | | | |
| 130 | Does the facility have a quality improvement (QI) team or committee responsible for improving the quality of services provided? | YES ............................................... 1  NO................................................ 2  DON’T KNOW.......................... 8 | 🡪201  🡪201 |
|  | IF YES, ASK: Could you describe this team/committee? Who participates? What does it do?  IF NO, ASK: Why not? | | |
| 131 | How many times in the past year has this team or committee met? | NEVER ......................................... 0  ONCE .......................................... 1  2-3 TIMES .................................. 2  4-5 TIMES .................................. 3  ≥ 6 TIMES .................................. 4  DON’T KNOW.......................... 8 | 🡪201 |
| 132 | How many times in the past year have staff discussed the quality of **nutrition** services provided? | NEVER ......................................... 0  ONCE .......................................... 1  2-3 TIMES .................................. 2  4-5 TIMES .................................. 3  ≥ 6 TIMES .................................. 4  DON’T KNOW.......................... 8 |  |

| **MODULE 4. WAREHOUSE INTERVIEW AND OBSERVATION** | | | | |
| --- | --- | --- | --- | --- |
| Now I would like to ask you about the availability of various supplies at this facility. I am interested in knowing about selected products you have in stock today and observing the general storage conditions. | | | | |
| 401 | Could you take me to the warehouse or room/building where nutrition products or supplies (e.g., specialized food products and micronutrient supplements) are stored? | YES .............................. 1  NO ............................... 2 | | 🡪 END |
| OBSERVE THE FOLLOWING ASPECTS OF THE WAREHOUSE OR ROOM/BUILDING WHERE NUTRITION PRODUCTS OR SUPPLIES ARE STORED. | | | | |
| 402 | STOREROOM IS MAINTAINED IN GOOD CONDITION (CLEAN, ALL TRASH REMOVED, STURDY SHELVES, ORGANIZED BOXES). | | YES ................................. 1  NO ................................. 2 | |
|  | COMMENTS: __________________________________________________________________________________________________  __________________________________________________________________________________________________________________ | | | |
| 403 | CURRENT SPACE AND ORGANIZATION IS SUFFICIENT FOR EXISTING PRODUCTS. | | YES ................................. 1  NO ................................. 2 | |
|  | COMMENTS: __________________________________________________________________________________________________  __________________________________________________________________________________________________________________ | | | |
| 404 | CARTONS AND PRODUCTS ARE IN GOOD CONDITION, NOT CRUSHED, WET, OR OTHERWISE DAMAGED DUE TO MISHANDLING. | | YES ................................. 1  NO ................................. 2 | |
|  | COMMENTS: __________________________________________________________________________________________________  __________________________________________________________________________________________________________________ | | | |

| NOW EXPLAIN: I am specifically interested in knowing the stock status of nutrition drugs/supplements for the period of [TIME PERIOD]; and today______, the day of the interview.   1. ASK: Is [PRODUCT] managed (typically stocked) at this facility? CIRCLE THE CODE “1” FOR “YES”, “2” FOR “NO”, OR “8” FOR “DON’T KNOW”.   FOR ALL PRODUCTS MANAGED AT THE FACILITY, ASK TO SEE THE STOCK CARD, ASK EACH QUESTION AND REVIEW STOCK CARDS AS INDICATED BELOW.   1. REVIEW THE STOCK CARD. CIRCLE THE CODE “1” IF THERE IS AN ENTRY FROM A SPECIFIED REPORTING PERIOD DETERMINED IN COUNTRY, “2” IF THERE IS NO SUCH ENTRY, OR “9” IF THERE IS NO STOCK CARD. 2. REVIEW THE STOCK CARD OR STOCK. CIRCLE THE CODE “1” IF THERE IS STOCK ON HAND (ANY QUANTITY), “2” IF THERE IS NONE, OR “9” IF THERE IS NO STOCK CARD. 3. ASK: Has the facility had a stock-out of [PRODUCT] during the [SPECIFIED REPORTING PERIOD]? CIRCLE THE CODE “1” FOR “YES”, “2” FOR “NO”, OR “8” FOR “DON’T KNOW”. | | | | |
| --- | --- | --- | --- | --- |
| **Product** | **A. Is [PRODUCT] managed at this facility?** | **B. OBSERVE: IS THERE AN**  **ENTRY IN THE STOCK CARD**  **FROM [SPECIFIED**  **REPORTING PERIOD]?** | **C. REVIEW STOCK CARD: IS STOCK OF [PRODUCT] ON HAND?** | **D. Have you had any stockout of [PRODUCT] in the [SPECIFIED REPORTING PERIOD]?** |
| 405. Folic Acid | YES ................. 1  NO ................. 2 🡪 Q406 DK .................. 8 🡪 Q406 | YES ................................................ 1  NO ................................................ 2  NO, STOCK CARD ................... 9 | YES ............................................... 1  NO................................................ 2  NO, STOCK CARD .................. 9 | YES ............................................... 1  NO ................................................ 2  DK ................................................. 8 |
| 406. Iron | YES ................. 1  NO ................. 2 🡪 Q407 DK .................. 8 🡪 Q407 | YES ................................................ 1  NO ................................................ 2  NO, STOCK CARD ................... 9 | YES ............................................... 1  NO................................................ 2  NO, STOCK CARD .................. 9 | YES ............................................... 1  NO ................................................ 2  DK ................................................. 8 |
| 407. Iron-folate tablets | YES ................. 1  NO ................. 2 🡪 Q408 DK .................. 8 🡪 Q408 | YES ................................................ 1  NO ................................................ 2  NO, STOCK CARD ................... 9 | YES ............................................... 1  NO................................................ 2  NO, STOCK CARD .................. 9 | YES ............................................... 1  NO ................................................ 2  DK ................................................. 8 |

|  | **Product** | YES ................. 1  NO ................. 2 🡪 Q409  DK .................. 8 🡪 Q409 | **B. OBSERVE: IS THERE AN**  **ENTRY IN THE STOCK CARD**  **FROM [SPECIFIED**  **REPORTING PERIOD]?** | **C. REVIEW STOCK CARD: IS STOCK OF [PRODUCT] ON HAND?** | **D. Have you had any stockout of [PRODUCT] in the [SPECIFIED REPORTING PERIOD]?** |
| --- | --- | --- | --- | --- | --- |
| 408. | Vitamin A | **A. Is [PRODUCT] managed at this facility?** | YES ................................................ 1  NO ................................................ 2  NO, STOCK CARD ................... 9 | YES ............................................... 1  NO................................................ 2  NO, STOCK CARD .................. 9 | YES ............................................... 1  NO ................................................ 2  DK ................................................. 8 |
| 409. | Multivitamins | YES ................. 1  NO ................. 2 🡪 Q410 DK .................. 8 🡪 Q410 | YES ................................................ 1  NO ................................................ 2  NO, STOCK CARD ................... 9 | YES ............................................... 1  NO................................................ 2  NO, STOCK CARD .................. 9 | YES ............................................... 1  NO ................................................ 2  DK ................................................. 8 |
| 410. | Albendazole | YES ................. 1  NO ................. 2 🡪 Q411 DK .................. 8 🡪 Q411 | YES ................................................ 1  NO ................................................ 2  NO, STOCK CARD ................... 9 | YES ............................................... 1  NO................................................ 2  NO, STOCK CARD .................. 9 | YES ............................................... 1  NO ................................................ 2  DK ................................................. 8 |
| 411. | Mebendazole | YES ................. 1  NO ................. 2 🡪 Q412 DK .................. 8 🡪 Q412 | YES ................................................ 1  NO ................................................ 2  NO, STOCK CARD ................... 9 | YES ............................................... 1  NO................................................ 2  NO, STOCK CARD .................. 9 | YES ............................................... 1  NO ................................................ 2  DK ................................................. 8 |
| 412. | IPT for malaria | YES ................. 1  NO ................. 2 🡪 Q413 DK .................. 8 🡪 Q413 | YES ................................................ 1  NO ................................................ 2  NO, STOCK CARD ................... 9 | YES ............................................... 1  NO................................................ 2  NO, STOCK CARD .................. 9 | YES ............................................... 1  NO ................................................ 2  DK ................................................. 8 |
| 413. | RUTF F-75 | YES ................. 1  NO ................. 2 🡪 Q414 DK .................. 8 🡪 Q414 | YES ................................................ 1  NO ................................................ 2  NO, STOCK CARD ................... 9 | YES ............................................... 1  NO................................................ 2  NO, STOCK CARD .................. 9 | YES ............................................... 1  NO ................................................ 2  DK ................................................. 8 |

|  | **Product** | YES ................. 1  NO ................. 2 🡪 Q415  DK .................. 8 🡪 Q415 | **B. OBSERVE: IS THERE AN**  **ENTRY IN THE STOCK CARD**  **FROM [SPECIFIED**  **REPORTING PERIOD]?** | **C. REVIEW STOCK CARD: IS STOCK OF [PRODUCT] ON HAND?** | **D. Have you had any stockout of [PRODUCT] in the [SPECIFIED REPORTING PERIOD]?** |
| --- | --- | --- | --- | --- | --- |
| 414. | RUTF F-100 | **A. Is [PRODUCT] managed at this facility?** | YES ................................................ 1  NO ................................................ 2  NO, STOCK CARD ................... 9 | YES ............................................... 1  NO................................................ 2  NO, STOCK CARD .................. 9 | YES ............................................... 1  NO ................................................ 2  DK ................................................. 8 |
| 415. | RUSF | YES ................. 1  NO ................. 2 🡪 Q416 DK .................. 8 🡪 Q416 | YES ................................................ 1  NO ................................................ 2  NO, STOCK CARD ................... 9 | YES ............................................... 1  NO................................................ 2  NO, STOCK CARD .................. 9 | YES ............................................... 1  NO ................................................ 2  DK ................................................. 8 |
| 416. | Dry rations (food) | YES ................. 1  NO ................. 2 🡪 Q417 DK .................. 8 🡪 Q417 | YES ................................................ 1  NO ................................................ 2  NO, STOCK CARD ................... 9 | YES ............................................... 1  NO................................................ 2  NO, STOCK CARD .................. 9 | YES ............................................... 1  NO ................................................ 2  DK ................................................. 8 |
